# The Radical Cure of Inguinal Hernia1Delivered at the Medical Graduates’ College.

**Published:** 1904-09

**Authors:** Mayo Collier


### CLINICAL LECTURE.

### The Radical Cure of Inguinal Hernia.[1]

By MAYO COLLIER, M. S., F. R. C. S.

[From *The Polyclinic* (London), August, 1904.]

GENTLEMEN—I have brought for your inspection today amongst other cases, one quite simple of diagnosis, but about which there is much to say and many points to discuss.

The patient as you see, is apparently a strong, healthy fellow, who comes to the hospital with the most absolute belief and faith that I can perform some simple operation on what he says is a rupture in the left groin, so that he will never have to wear a truss again, and never be troubled with a return of his complaint. In short, he wants me to perform the radical cure in the belief that the radical cure will do for him all that he thinks.

This is the general belief among the lay public on the subject of the operation for the relief of rupture. I need hardly tell you that this view is incorrect; and the approximate truth on the subject of the radical cure for hernia I shall endeavor to put before you today.

Now, the first question you must be prepared to answer is this: Why an operation at all? Why not a comfortable and well-fitting truss? This question must be answered from several points of view, and, firstly, there are seven classes of cases where a truss cannot be relied upon, and an operation is most certainly indicated, namely: (1) cases of irreducible hernia; (2) cases of strangulated hernia; (3) cases where the hernia is not controlled by a truss; (4) cases of hernia with ectopic testis; (5) cases where rupture unfits for the public service; (6) hernias in incompetent and ignorant people; (7) very large hernias hampering the movements and seriously threatening the personal comfort of the patients.

In all these cases it would be your duty to prefer an operation rather than outside mechanical supports. In other cases it is quite certain that hundreds and thousands of persons pass through their lives with comfort and safety with the assistance of a well-fitting truss, but I most certainly would not refuse to perform the radical cure for a person who was anxious to be relieved of his truss, and who, from a sentimental point of view, objected to the mechanical support.

Now, before you could honestly take upon yourself the responsibility of advising and operating on a patient for the cure of hernia, you must satisfy yourself, and in some cases your

1. Delivered at the Medical Graduates' College.

patient, as to the following facts, and you must be prepared with more or less exact knowledge on the subject:

(1) Can you assure him that the operation is a safe operation so far as his life is concerned, as well as free from risk of damage to his testicles?

(2) Does the term "radical cure" mean radical cure in the sense of permanency of cure? Is the operation a real cure or only a temporary palliative?

(3) Should very young people be operated upon? If not, within what age-limits should the operation be performed?

(4) Should a truss be worn after the operation? If so, for how long, and, if not, should any support or precaution be taken to prevent return?

(5) You must satisfy yourself as to the best material for suture, and, lastly, but assuredly not least, the best form of operation to perform.

You see from this list of questions that you must answer either to the patient or yourself, that the procedure is not quite so simple as the public believe, and not quite so lightly to be undertaken as some would have us teach.

And, first of all, as to the safety of the operation. I think we may most certainly answer this question in the affirmative and say "the operation is perfectly safe, provided it is done by a skilled and competent operator with experience."

Out of 104 cases in one practice there were only two deaths, both from zymotic diseases, and, in America, Dr. Coley, out of 160 operations, lost one case, from pneumonia.

These are just a few examples illustrating the freedom from trouble after this operation.

Next, is the testis in any danger from the operation? It most certainly is in the hands of the unskilled surgeon who endeavors to pull and drag and rend the layers of fascia covering the hernial sac, instead of cutting straight down to the sac and gently peeling it from its nearest covering.

Atrophy of the testis, and inflammation with abscess and sloughing, have followed this operation in careless hands.

Next we come to the question, How far does the operation bring about a complete or radical cure? Also, how far is this cure a permanent one? Please always safeguard your own reputation, and that of surgery in general, by telling your patients that many of the best and most promising cases lapse—that the hernia returns and requires a second operation or a truss. Tell your patient that only in some cases does the patient never require further assistance or treatment, but also tell your patient "that an operation will most certainly improve matters, that after an operation a patient is better off than he was before, and that most certainly it gives immunity to a certain number for a certain time."

As a matter of fact, it is fair to say that in 25 per cent. of cases operated on the hernia returns and requires a second operation or the assistance of a truss before eight years.

The next question you have to settle is, What is the earliest age that an operation should be undertaken?

It is a matter of common knowledge that quite a number of male children below one year have inguinal hernia, and that quite a large percentage of these get well with the use of properly-fitting trusses.

On these grounds a truss should in most cases be recommended in preference to an operation up to, we will say, the fourth year, and in some cases longer. Of course, complicated cases where there are no contraindications should be submitted to operation as early as two years.

The next point that will engage your attention is the desirability or otherwise of using a truss subsequent to operation.

This recommendation will depend not only on the state of the patient before operation, but on his condition and duties after the operation. Speaking generally, favorable cases with small sacs, sound tissues, and well-formed abdominal walls, if allowed to rest for at least two or three months after operation, will not require a truss. *Per contra,* large sacs, weak abdominal walls, pendulous abdomens with much fat are better supported with a broad, flat truss. The same applies to all cases where, after operation, the patient has to perform heavy and arduous work, or suffers from chronic bronchitis, asthma, or chronic engorgement of the portal system.

Again, patients going abroad, where skilled surgical assistance is not available, are better to be provided with the safeguard of a light, broad, well-fitting truss, to be worn on all occasions when in the erect position.

A point of considerable importance in the after-care of patients who have undergone the radical cure has been completely overlooked, so far as I know, by all writers on this subject. It is this —the method and position and preparation for the act of defecation. "Straining at stool"—how often have we who have been house surgeons or those in large practices heard this suggested by the patient afflicted with rupture as the direct cause of the accident. The act of defecation as nature intended us to perform the same is in the natural position with the extremities and trunk all flexed to an acute angle—the thighs flexed on the abdomen so that the knees shall be in contact with the chest, the back of the leg touching the ham and the arms folded round the knees. No pressure that was ever exerted in this attitude could affect the inguinal or femoral openings. They are completely shut off and supported.

The modern artistic W. C. imposes the necessity of sitting with the body erect and the thighs at a right angle to the trunk. In this attitude the inguinal and femoral openings stand the whole pressure of the abdominal contents in straining during defecation.

Therefore, after, if not before the operation for radical cure, direct your patient to return, so far as the act of defecation is concerned, to the primitive style and method. There is no doubt that the civilised position of the act of defecation is responsible for a large percentage of ruptures. I most certainly should advise all persons who have a tendency to hernia or weak abdominal walls to stand upon the seat of the W. C., not sit upon it—this is a point of the utmost importance in the prevention and cure of hernia.

The next question is—What is the best material for suture and ligature, and more especially for the ligature of the neck of the sac? Every surgeon of any experience must have been disappointed to find some five or six weeks after the operation that his patient presents himself with a small discharging sinus in the site of the ligature surrounding the neck of the sac; this is the recorded experience of some of the best surgeons of the day when prepared silk has been used.

Many substances have been recommended—catgut, kangaroo tail, silkworm's gut, silver wire and Chinese plaited silk, such as is used for fishing lines. The ideal ligature of course is one that is perfectly reliable, strong, easily rendered aseptic, and will be innocuous and nonirritating to the tissues.

Each of the above in turn has been tried and each has its advocates, but for general use, ease of application, freedom from becoming undone, and reliability, plaited Chinese silk seems to be the favorite. American surgeons speak most highly of kangaroo tail, others use silver wire, and many Colonial surgeons prefer catgut. The feeling of security that Chinese silk affords at the time of operation, as well as the experience of its use in other parts of the body, more especially in the suture of tendons, has lent some considerable support to its use in this operation, but still this ligature is constantly the cause of suppuration weeks and months after the operation, delaying convalescence and bringing discredit on the operation and the surgeon who performs it. The cause of this is not apparent.

The same ligature placed on a tendon or round the femoral artery would not suppurate. It may be due to the fact that the site of ligature is in relation to the larger bowel whence foul gases may exhale and pass into the tissue, thus rendering this lifeless ligature septic, whereas a living structure would resist the infection. To sum up, this accident is liable to occur, and for the operation a perfect ligature has yet to be found.

Speaking of the other ligatures. Kangaroo tail is difficult to obtain. Catgut is liable to soften too easily and be absorbed too soon, and wire cuts through and is liable to prove painful and irritating.

The last and most important point to settle is the choice of operation. You will either admire the ingenuity of man, or deplore the unsettled state of surgical opinion, when I tell you that there are at least ten well-recognised surgical procedures for the relief of rupture in the inguinal region.

You will pardon the liberty I am taking when I say that no man who is not perfectly at home with, and acquainted with, the anatomy of this region in every detail should undertake this operation.

With your permission I will just remind you of a few salient points in the anatomy of this region. And first of all this rupture is situated in the inguinal canal. Now, what is the inguinal canal?

It is an oblique passage, some two and a half inches in length, through the layers of the lower mesial part of the abdominal wall. Commencing in the deeper layers of the wall, at the so-called deep abdominal ring, it passes downwards and inwards towards the middle line, near to which it has its superficial opening, the superficial abdominal ring. The deep abdominal ring is situated midway between the anterior superior spinous process of the ilium and the spine of the pubes, and one inch above Poupart's ligament. It is oval in shape and measures about three-quarters of an inch from above downwards and half-an-inch from side to side. Now, what is this ring in its essence? It is formed by the margin of junction of the infundibuliform fascia with the transversalis fascia exactly in the same way as where the finger of a glove joins the body of the glove. It is covered over on its deep aspect by peritoneum with subperitoneal fat and gives passage to the spermatic cord with its arteries, veins, and nerves, these being, of course, outside the peritoneum.

The superficial and mesial limit of the inguinal canal is the superficial abdominal ring. This is situated immediately above the spine of the pubes. It is triangular in shape with its base below and its apex pointing upwards and outwards. It is about one and a quarter inches from base to apex and less than an inch across its base. The two sides of the triangle are known as the pillars of the ring. In order properly to understand the anatomy of the pillars of the ring an exact knowledge of the formation and attachment of Poupart's ligament is requisite. Poupart's ligament is the lower thickened border of the aponeurosis of the external oblique muscle. It is attached externally to the anterior superior spinous process of the ilium, and internally has three attachments, which please note carefully, as the key to the surgery of both femoral and inguinal hernia rests with a knowledge of these. The three attachments are:

(*a*) To the spine of the pubes forming the external pillar of the ring.

(*b*) To the ilio-pectineal line for about one and a quarter inches forming Gimbernat's ligament.

(*c*) To the linea alba forming the triangular ligament or fascia known as Coll's ligament.

Practically, speaking, after Poupart's ligament has become attached to the spine of the pubes it spreads in two directions, horizontally to form Gimbernat's ligament, and vertically, to form Coll's ligament.

Now we are in a position to refer again to the superficial abdominal ring with its pillars. The external pillar of the ring is formed by Poupart's ligament, it is round and cord-like and nearly horizontal in position. The internal pillar of the ring is thin, tendinous, straight and sharp, and is formed by fibres of the tendon of the external oblique that pass to the front of the pubes and here interlace with those of the opposite side.

The base of the triangle is formed by the crest of the pubes and extends from the spine of the pubes to the symphysis pubis. The apex of the triangle is acute and is crossed by well marked fibres, passing from one pillar to the other, and known as intercolumnar fibres. Having dealt with the two extremities of the passage, we now come to the boundaries and structures forming the passage itself, and, in order to do so, I will just remind you of how the abdominal muscles comport themselves in this region.

You will remember the general disposition of these three muscles. Two are oblique and one is transverse. The superficial one (external oblique) is oblique from above downwards and the next in order (internal oblique) is oblique from below upwards and inwards, and the deepest layer (transversalis muscle) has its fibers running almost transversely. The upper attachment of these muscles does not concern us. We have seen how the external oblique bridges over the groin in the form of a thickened band of fibrous tissue known as Poupart's ligament, and how this is attached externally to the superior spinous process of the ilium, and internally to the spine of the pubes and ilio-pectineal line and linea alba.

The internal oblique also arches over the groin in the same way as the external oblique, but only towards the inner half of the groin from the inner third of Poupart's ligament to the ilio-pectineal line. Its outer fibers are thick and muscular, and are attached to the outer two-thirds of Poupart's ligament, and, after covering the outer half of the inguinal canal, dip downwards and inwards to be inserted into ilio-pectineal line internal to Poupart's ligament, and posterior to the inner half of the inguinal canal. The internal oblique then is anterior to the outer half of the canal and posterior to the inner half.

The transversalis muscle also arches at its lower border over the groin. It has much the same disposition as the internal oblique, but, having a much less extensive attachment to Poupart's ligament (only to its outer third), passes inwards and arches downwards to join the tendon of the internal oblique, and to be inserted with it into the ilio-pectineal line, forming the conjoined tendon about which you will hear so much during the operation for the radical cure of hernia. The transversalis muscle, passing above the deep ring, has no anterior relation to the inguinal canal, but, in passing to its attachment as the conjoined tendon, is in a posterior relation to the inner half of the canal. On the deep surface of the transversalis muscle we have the general lining membrane of the abdomen just as an egg has its general fibrous lining internal to the shell. This membrane receives various names in various places. When lining the transversalis muscle it is known as the transversalis fascia. In the pelvis it is known as the pelvic fascia, and that portion lining the diaphragm would be known as the diaphragmatic fascia. The deep abdominal ring is an opening in this fascia caused by the bag-like pouch that is carried down by the testicle on leaving the abdominal cavity.

Well, then, we are now completely in possession of the facts in the anatomy of these parts that relate to the operation, and we know that the canal has only in front of it the external and internal oblique muscles, and behind it the internal oblique muscle and transversalis muscle in the form of the conjoined tendon, and behind this the transversalis fascia.

You know also that the deep epigastric artery runs upwards and inwards from the iliac artery, and, passing between the transversalis fascia and peritoneum, skirts close to the inner margin of the deep ring. In operating you must always keep the exact position of the artery in mind.

An oblique inguinal hernia, then, is a protrusion of any of the abdominal contents through the entire length of the inguinal canal and would have for its covering representatives of each of the normal layers of the abdominal wall in anterior relation to the canal. First we should have skin, and then superficial fascia, then external oblique represented by the inter-columnar fascia, then internal oblique or its representative the cremasteric fascia, then transversalis or infundibuliform fascia, and, lastly, superitoneal fat and peritoneum. The transversalis muscle not being in anterior relation to the canal escapes being pushed in front of the descending testis.

These few anatomical facts having just refreshed our memories, we are now in a position to undertake the operation for the radical cure of hernia.

Now, of the ten surgical procedures I forshadowed to you, all have the same object in view—namely, to get rid of the sac of peritoneum up to and as far as the deep abdominal ring, and to close the passage through the layers of the abdominal wall by readjusting these.

These objects are attained by various methods. The lecturer then related and described the ten operations for the radical cure of inguinal hernia, and expresed a personal predilection for Mr. Barker's operation.

